# Significant contribution of the unicellular cyanobacterium UCYN-B to oceanic nitrogen fixation

**DOI:** 10.1093/nsr/nwaf337

**Published:** 2025-08-14

**Authors:** Ruotong Jiang, Haizheng Hong, Zuozhu Wen, Xinran Yu, Thomas J Browning, Ze Chen, Yanpeng Shang, Xin Liu, Zhimian Cao, Eric P Achterberg, Minhan Dai, Dalin Shi

**Affiliations:** State Key Laboratory of Marine Environmental Science, Xiamen University, Xiamen 361102, China; State Key Laboratory of Marine Environmental Science, Xiamen University, Xiamen 361102, China; State Key Laboratory of Marine Environmental Science, Xiamen University, Xiamen 361102, China; State Key Laboratory of Marine Environmental Science, Xiamen University, Xiamen 361102, China; Marine Biogeochemistry Division, GEOMAR Helmholtz Centre for Ocean Research Kiel, Kiel D-24148, Germany; State Key Laboratory of Marine Environmental Science, Xiamen University, Xiamen 361102, China; State Key Laboratory of Marine Environmental Science, Xiamen University, Xiamen 361102, China; State Key Laboratory of Marine Environmental Science, Xiamen University, Xiamen 361102, China; State Key Laboratory of Marine Environmental Science, Xiamen University, Xiamen 361102, China; Marine Biogeochemistry Division, GEOMAR Helmholtz Centre for Ocean Research Kiel, Kiel D-24148, Germany; State Key Laboratory of Marine Environmental Science, Xiamen University, Xiamen 361102, China; State Key Laboratory of Marine Environmental Science, Xiamen University, Xiamen 361102, China

**Keywords:** nitrogen fixation, unicellular diazotrophic cyanobacteria, UCYN-B, ocean productivity

## Abstract

Dinitrogen (N_2_) fixation by diazotrophs forms a critical control on ocean productivity, yet our understanding of the distribution, abundance and activity of these microorganisms is informed by observations with limited geographical coverage. Here we show that the unicellular N_2_-fixing cyanobacterium, UCYN-B, drives high N_2_ fixation rates in the western North Pacific. Defining the ecological niche of UCYN-B using our new observations in combination with existing global diazotroph datasets allowed prediction of additional and previously unrecognized UCYN-B-dominated N_2_ fixation hot spots throughout large, unsampled swathes of the global ocean. Collectively, these regions potentially contribute 10.8–15.0 Tg N yr^−1^ to global marine N_2_ fixation, suggesting a pivotal role for UCYN-B in the regulation of the marine nitrogen inventory and therefore ocean productivity.

## Teaser

UCYN-B drives high N2 fixation rates in previously unrecognized hot spots across the global ocean, making a significant contribution to the marine nitrogen inventory and ocean productivity.

## INTRODUCTION

Primary production throughout most of the ocean is limited by the availability of fixed nitrogen (N) [1], which is ultimately determined by the balance between input and loss processes [2]. N_2_-fixing microorganisms (diazotrophs) convert N_2_ gas into ammonium, which together with other minor N sources (e.g. aerosols and rivers) balances N losses via denitrification and anammox [3]. Whether the oceanic N budget is in balance is both critically important to the Earth system and highly dependent on accurately measured oceanic N_2_ fixation rates [4]. Both N_2_ fixation rates and diazotroph abundances remain strongly under-sampled; diazotroph ecological niche assessment (environmental conditions associated with different diazotroph species) provides a mean to make much wider projections based on environmental predictors [5]. The filamentous cyanobacterium *Trichodesmium* and the symbiotic cyanobacterium *Richelia* have historically been considered to be the dominant diazotrophs in warm, low N (sub)tropical waters [6–9]. More recently, the unicellular diazotrophic cyanobacterium UCYN-A has been shown to be widely distributed in the oceans, particularly in colder, N-rich coastal upwelling and higher latitude regions [10–12]. Accordingly, these niches have constrained environmentally-driven predictions of marine N_2_ fixation [5]. Therefore, the discovery of new niches has the potential to broaden spatial-temporal predictions of global marine diazotrophy.

Until recently, our understanding of diazotrophy in the North Pacific Subtropical Gyre (NPSG), one of Earth's largest biomes, has been primarily informed by studies at the Hawaii Ocean Time-series site. Here, UCYN-A has been found to dominate diazotrophic communities [13,14], alongside frequent *Trichodesmium* and *Richelia* blooms in summer [15,16]. More recent observations have shown considerable variability in both N_2_ fixation rates and diazotroph community structure across the NPSG, accompanied by large spatial gradients in the physical and biogeochemical characteristics of seawater [17–21]. For example, Wen *et al.* [21] observed high abundance of the unicellular cyanobacterium UCYN-B in the diazotroph community and high N_2_ fixation rates along an ∼3000 km section in the (sub)tropical western North Pacific. Machine learning models based on high-resolution observations further underscore the global significance of N_2_ fixation in this region [22], where diazotrophy has been under-sampled and high rates of N_2_ fixation have not been widely observed [5,23]. While the N_2_ fixation capacity of UCYN-B has been demonstrated through both field observations and laboratory cultures [24,25], there has been limited documentation of its elevated abundances in the global ocean, with a few reports restricted to the station ALOHA and a limited area of the South Pacific Gyre [26–28]. In fact, the global distribution and regulatory mechanisms of UCYN-B are not yet well known [29], and its contribution to global N_2_ fixation may have been overlooked mainly due to lack of investigation.

In this study, we systematically evaluated the importance of UCYN-B to global oceanic N_2_ fixation by first conducting two additional large-scale (∼11 000 km) field sampling campaigns in the western North Pacific in both summer and winter (Fig. S1); second, assessing the ecological niches of UCYN-B alongside the other three major cyanobacterial diazotrophs (i.e. *Trichodesmium*, UCYN-A and *Richelia*) using Generalized Additive Models (GAMs) with our new observations in combination with recently reported global databases of diazotrophy [30]; and finally, estimating the biogeography and contribution of UCYN-B to N_2_ fixation in the global ocean.

## RESULTS AND DISCUSSION

### UCYN-B dominated diazotroph community in the western North Pacific

**Figure 1.**
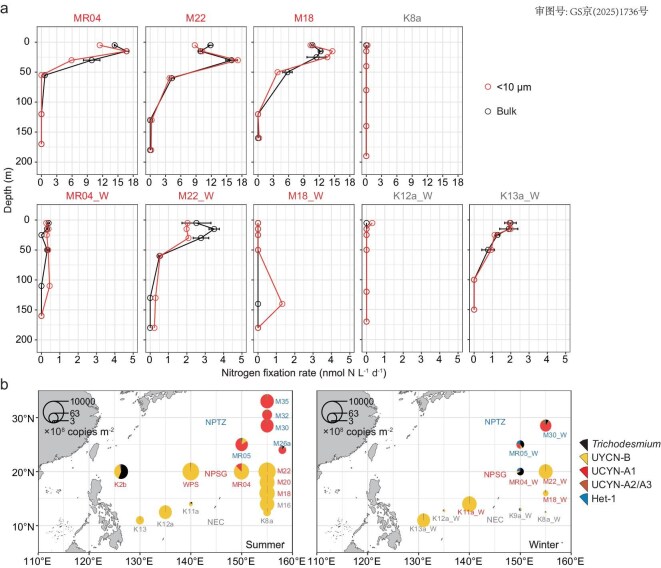
Distribution of N_2_ fixing diazotrophs in the western North Pacific. (a) Depth profiles of N_2_ fixation rates in bulk-water (mean values ± SD, *n* = 2 biologically independent samples) and <10-μm size fraction (*n* = 1) of the diazotroph community in NPSG and NEC stations, and (b) depth-integrated *nifH* gene abundance of five major cyanobacterial diazotrophs in the western North Pacific in summer and winter. The study area was divided into three regions, i.e. the central area of the North Pacific Subtropical Gyre (NPSG, red), the North Pacific Transition Zone (NPTZ, blue) and the North Equatorial Current affected area (NEC, grey).

Depth-integrated N_2_ fixation rates for the NPSG stations in our study ranged between 199 and 821 μmol N m^−^^2^ d^−^^1^ in summer and declined to 20 and 228 μmol N m^−^^2^ d^−^^1^ in winter (Fig. S1). These high rates are comparable to reported rates in this area in late spring (429 ± 93 μmol N m^−2^ d^−1^) [21], and are amongst the highest rates reported in the global ocean (Table 1 and Fig. S1). These results demonstrate high levels of N_2_ fixation in the western (sub)tropical North Pacific. At the NPSG and adjacent North Equatorial Current (NEC) affected stations with high N_2_ fixation rates (e.g. MR04, M22, M18 and K13a_W; see Table S1 for station information), the <10 μm size fraction of the diazotroph community accounted for 63%–98% of bulk N_2_ fixation in both summer and winter (Fig. 1a), which is in line with previous studies showing that nanoplanktonic cyanobacteria were the major diazotrophs in this region, especially in summer [18]. Metagenomic analyses showed that UCYN-B accounted for >65% of the diazotroph community at stations WPS and M22 in summer where high N_2_ fixation rates were observed (Table S2). The *nifH* gene amplicon sequencing further revealed that >70% of the DNA sequences at stations with high N_2_ fixation rates (K2b, WPS, MR04, M22, M22_W and K11a_W) were identical to UCYN-B (Fig. S2a, b), and showed 99%–100% nucleotide similarity to *Crocosphaera watsonii* WH8501 (Fig. S2c). Quantitative analysis of the absolute *nifH* gene abundance demonstrated that UCYN-B constituted the major portion (67%–99%) of the total depth-integrated *nifH* gene abundances of the major N_2_-fixing cyanobacterial diazotrophs at the NPSG and NEC stations with high N_2_ fixation rates in both seasons, except for K2b where a *Trichodesmium* bloom occurred during sampling (Fig. 1b).

**Table 1.** Areal N_2_ fixation rates in the Northwest (Sub)tropical Pacific and the identified hot spots of N_2_ fixation in the global ocean.

Regions	Areal N_2_ fixation rate (μmol N m^−2^ d^−1^)^a^	Number of observations
**North Pacific**		
Western NPSG (summer)^b^,^c^	593 ± 233	7
Western NPSG (winter)^b^,^c^	102 ± 95	4
Western NPSG (spring) [21]^c^	429 ± 93	7
Kuroshio (summer) [20]^d^	239 ± 84	3
Kuroshio intrusion-affected South China Sea (summer) [31]^c^	463 ± 260	6
ALOHA (annual) [32]^c^,^d^	228 ± 135	71
**Tropical South Pacific**		
Bismarck Sea (austral summer) [33]^c^	415 ± 218	6
North Solomon Sea (austral summer) [33]^c^	2410 ± 1869	6
Melanesian Archipelago (austral summer) [34]^d^	631 ± 286	17
New Caledonia (annual) [34]^d^	301 ± 208	5
**North Atlantic**		
Tropical North Atlantic (annual) [35]^d^,^e^	239 ± 38	154
Tropical North Atlantic (summer) [36]^c^	305 ± 277	14

a Mean value ± standard deviation; ^b^Stations assigned to NPSG (North Pacific Subtropical Gyre) are shown in Fig. 1; ^c^N_2_ fixation rates measured using the ^15^N_2_-enriched water method; ^d^N_2_ fixation rates measured using the ^15^N_2_ gas method; ^e^N_2_ fixation rates measured using the C_2_H_2_ reduction method.

Evidence for the importance of UCYN-B in driving high N_2_ fixation rates in the North Pacific is limited [26,28,37], with reports instead suggesting *Trichodesmium* and UCYN-A as the most important phylotypes [6,13,14,17,24]. This may be a result of previous investigations being largely restricted to either western boundary regions [38–40] or the Hawaiian Ocean Timeseries site [13,16,17,41]. Among the diazotrophic phylotypes we surveyed by quantitative *nifH* gene analysis, UCYN-B explained most of the variability of, and showed consistent spatial and temporal patterns with, measured bulk N_2_ fixation rates (*R* = 0.751 and *P* < 0.001; Fig. 1b and Figs S1 and S3). Furthermore, by using the reported average cell-specific N_2_ fixation rates of UCYN-B measured by nanometer-scale secondary ion mass spectrometry (nanoSIMS) [42–44], and an average UCYN-B *nifH* gene polyploidy copy number [26,45] (Note S1 and Table S3), we estimated that UCYN-B could contribute ∼90% (71.9%–115.1% 95% confidence interval) of the measured N_2_ fixation rates in the NPSG and NEC regions (Fig. S4 and Table S4). In contrast, we found *Trichodesmium* was only prevalent in regions near the western boundary of the North Pacific (station K2b), and UCYN-A was dominant at the North Pacific transition zone (NPTZ), north of the NPSG, where N_2_ fixation rates were relatively low (Fig. 1b, Table S2 and Fig. S1).

In addition to cyanobacterial diazotrophs, non-cyanobacterial diazotrophs (NCDs) have been found to be widely distributed in the global ocean, including the eastern Pacific Ocean [23,46–48]. Recent studies have reported N_2_ fixation by putative particle-attached NCDs in oxygenated surface waters [49], as well as by non-cyanobacterial N_2_-fixing symbionts in the tropical North Atlantic [50]. In our study, using metagenomic analysis and *nifH* gene amplicon sequencing, we found that Alpha- and Gamma-proteobacterium were the two main NCDs in the western North Pacific (Table S2 and Fig. S2). However, these NCDs were only dominant at stations where N_2_ fixation rates declined significantly (e.g. MR04_W) or were not detectable (e.g. K8a, K11a and K8a_W) (Figs S1 and S2).

Therefore, our study fills a major observational gap, and suggests that UCYN-B is primarily responsible for both the high overall diazotroph abundance and N_2_ fixation rates in the under-sampled western North Pacific, particularly in the NPSG and NEC regions [5,23].

### Ecological niche and distribution of UCYN-B in the global ocean

**Figure 2.**
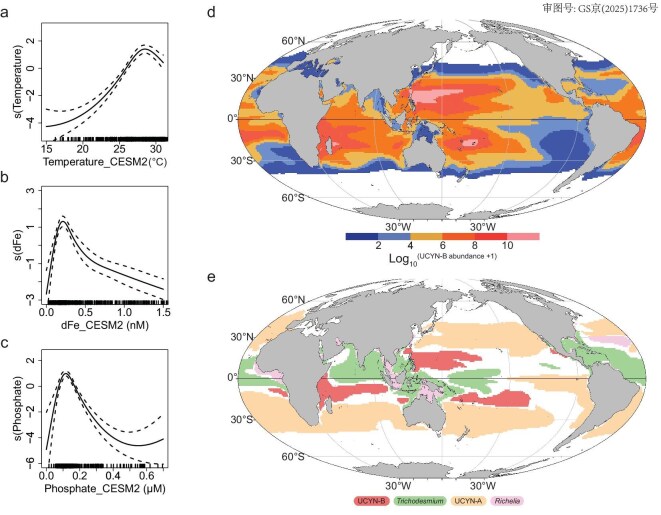
Ecological niche and distribution of UCYN-B in the global ocean. (a–c) GAM-modeled response curves of UCYN-B against (a) surface temperature (SST), (b) dissolved Fe (dFe) and (c) phosphate (P) concentrations [Log_10_ (*nifH* gene abundance) ∼s(SST) + s(dFe) + s(P), (*n* = 605)]. UCYN-B appeared to correlate with all variables in the three-variable-GAM (*P* < 0.001). Our new observations were incorporated into a global database of depth-integrated *nifH* gene abundances [30], and used to conduct GAM analysis (Fig. S5, Note S2). The small dashes on the X-axis of (a) to (c) are empirical density distributions of observational covariates. (d) Predicted global distributions of UCYN-B *nifH* abundances using 10-year averaged monthly data for SST, dFe and P in CMIP6-CESM2 (re-gridded to a standard 1° × 1°). (e) The dominant regions of UCYN-B, UCYN-A, *Trichodesmium* and *Richelia* in the global ocean. The regions were defined as where the diazotroph type contributed to >75% of the total *nifH* gene abundance of the four diazotrophs surveyed and the total *nifH* gene abundance was higher than 10^4^ copies m^−^^2^.

To explore the potential global distribution of UCYN-B, our new observations were incorporated into a recently updated global dataset of depth-integrated *nifH* gene abundances [30], and used to evaluate the ecological niche of UCYN-B using GAMs. In total, 737 observations of depth-integrated *nifH* gene abundances in the global ocean were compiled (Fig. S5), and used in GAMs to estimate diazotroph niches along gradients of three key variables, i.e. sea surface temperature (SST), surface dissolved Fe (dFe) and surface dissolved phosphate (P) concentrations. These variables have been recognized as keys for regulating the distribution of major marine cyanobacterial diazotrophs globally (Note S2) [5,51]. In our analysis, the three parameters explain 54.9% (*R*^2^ = 0.539) of the variations in depth-integrated *nifH* gene abundance of UCYN-B, and all individually show significant relationships with UCYN-B (*P* < 0.001; Fig. 2a–c and Table S5). Significant correlations between SST, dFe and P and *nifH* gene abundance were also found for *Trichodesmium*, UCYN-A and *Richelia* (Fig. S6 and Table S5).

The temperature response curve of UCYN-B was characterized by a high temperature niche with a relatively narrow range (25–30°C), and below ∼26°C the abundance of UCYN-B declined rapidly (Fig. 2a). These results agreed with experimentally observed optimal growth temperatures for UCYN-B [52,53]. The temperature response curve for *Trichodesmium* was similar to that of UCYN-B (Fig. S6), implying that these two species may share a similar latitudinal niche in oligotrophic (sub)tropical regions [5,54]. In contrast, UCYN-A and *Richelia* had broader niches at lower temperatures (Fig. S6), in line with observations of UCYN-A extending to temperate and polar regions [5,10].

We found that UCYN-B correlated non-linearly with dFe concentrations, declining sharply at dFe concentrations below 0.2 nM (Fig. 2b). At dFe concentrations above 0.2 nM, UCYN-B showed a much narrower niche than *Trichodesmium* and *Richelia* (Fig. 2b and Fig. S6). The different dFe niches for unicellular and larger-sized, filamentous-forming diazotrophs may be related to their respective Fe utilization strategies. For example, in addition to having a higher surface-to-volume ratio that favors Fe uptake [55,56], unicellular species such as UCYN-B may be more competitive in regions with lower dFe concentrations via their ability to move Fe between cellular components over day and night cycles [57].

The P response curve for UCYN-B was similar to dFe, peaking at low P concentrations (∼0.1 μM) and declining sharply at higher P concentrations (Fig. 2c). This behavior was also observed for the other three diazotrophs (Fig. S6), and probably results from competition by faster growing non-diazotrophs in regions with simultaneously elevated N supply [58]. However, at lower P concentrations the decline in UCYN-B was much more pronounced than that of larger-size diazotrophs, potentially implying that unicellular diazotrophs are mainly reliant on the supply of dissolved inorganic phosphorus. This observation is consistent with reports that UCYN-B has a weaker capability to use dissolved organic phosphorus than *Trichodesmium* [59,60]. This trend could also result from a co-variability of the low P regions with high dFe regions, where *Trichodesmium* dominates.

The GAMs in our study clearly showed the distinct niches of UCYN-B (and the other three major cyanobacterial diazotrophs) in the context of the variability in SST, dFe and P (Fig. 2a–c and Fig. S6). We thus employed the relationships produced by the GAMs to predict the global distribution of UCYN-B *nifH* abundances, using 10-year averaged data of monthly SST, dFe and P from an ocean biogeochemical model simulation (CMIP6-CESM2, Note S3). UCYN-B was generally restricted to tropical and subtropical oceans, and the abundances increased significantly toward the west of each oceanic region except the North Atlantic (Fig. 2d). Our GAMs-based projections generally agreed well with the data-driven model simulations [5], as all the four major cyanobacterial diazotrophs showed similar niches projected by the two different approaches. However, a major difference between our projections and previous simulations was that UCYN-B was more abundant (2–3 orders of magnitude higher) in our projections, especially in the (sub)tropical western Pacific Ocean and western Indian Ocean [5] (Fig. S7), where the diazotroph communities have previously been predicted to be dominated by *Trichodesmium* or by multiple species [4,5]. The difference was largely due to the increased observations in the global ocean [30] and the unprecedentedly high UCYN-B abundances in the western North Pacific as shown in this and a previous study [21], which allowed for a more accurate UCYN-B niche analysis and thus global projection.

### UCYN-B driven hot spots of N_2_ fixation in the global ocean

**Figure 3.**
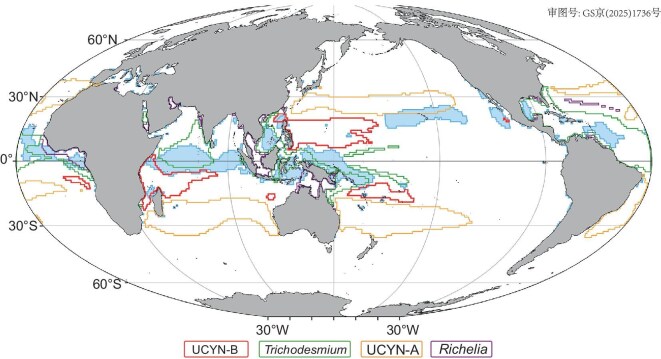
UCYN-B dominant regions with potential high N_2_ fixation rates. The delineated areas represent regions with total *nifH* gene abundance higher than 10^8^ copies m^−^^2^. Red, green, orange and purple denote regions dominated (defined as >75% of the total *nifH* gene abundance) by UCYN-B, *Trichodesmium*, UCYN-A and *Richelia*, respectively. The blue shaded areas denote regions predicted to have N_2_ fixation rates >100 μmol N m^−^^2^ d^−^^1^, the lower threshold of the high N_2_ fixation category in the global database [41,63,64].

Our observational and model results showed a previously unrecognized global dominance of UCYN-B, and its important contribution to N_2_ fixation in hot spots such as the (sub)tropical western North Pacific (Table 1 and Fig. 2d, e). These new findings imply that N inputs by UCYN-B may be of crucial importance in sustaining productivity in these regions as well as balancing oceanic N loss. To further assess this, we identified the areas with potentially high N_2_ fixation rates in UCYN-B dominant regions (>75% of total *nifH* gene abundance), including the tropical regions of the western North Pacific, western South Pacific Gyre, western Indian Ocean and eastern South Atlantic Gyre, using a minimal total *nifH* gene abundance threshold of 10^8^ copies m^−^^2^ (Fig. 3). Except for the western North Pacific which has been better characterized by this study and a previous study [21], assessment of N_2_ fixation rates and diazotroph abundances in these identified areas are limited (Fig. S8a), particularly in the Indian Ocean and the eastern South Atlantic. However, in the western South Pacific Gyre, high UCYN-B *nifH* gene transcript abundance (up to 10^11^ copies m^−2^) [61] and dominance of UCYN-B have been found, matching up with the region where we predict UCYN-B to dominate in a high N_2_ fixation rate area (Fig. S8b). In addition, the eastern South Atlantic Gyre has recently been identified as a hot spot of N_2_ fixation [62]. Finally, metagenomic results from Tara Oceans datasets indicate a high percentage (58%–84%) of UCYN-B in the cyanobacterial diazotroph community in the 0.8–5.0 μm size fraction at two surface stations (i.e. Tara-50 and Tara-51) around Madagascar in the South Indian Ocean [23]. Importantly, we found that none of these predicted UCYN-B dominated region areas overlapped with any regions previously predicted to have N_2_ fixation rates higher than 100 μmol N m^−^^2^ d^−^^1^ (Fig. 3), the designated lower threshold of the high N_2_ fixation category in the global database [41,63,64]. However, the potentially high-rate areas of *Trichodesmium* dominant regions (e.g. the tropical North Atlantic, western boundary of the Pacific Ocean and northern Indian Ocean) matched most of the previously modeled high N_2_ fixation regions [41,63]. These results emphasize (i) the reliability of the GAMs in projecting the diazotroph niches and distributions, and (ii) that UCYN-B's niche and the capability of this group to drive high N_2_ fixation rates were likely being missed in previous predictions due to limited field sampling.

Our new N_2_ fixation observations from the western North Pacific in this study were incorporated into the global N_2_ fixation database [30], to re-evaluate the total N_2_ fixation flux in the UCYN-B dominated, high N_2_ fixation rate area (*nifH* >10^8^ copies m^−^^2^ and N_2_ fixation >100 μmol N m^−^^2^ d^−^^1^) in the western North Pacific. The calculated mean rates in this area were 112 and 192 μmol N m^−^^2^ d^−^^1^ for geometric and arithmetic means (Table S6), respectively, higher than 87 and 177 μmol N m^−2^ d^−1^ estimated from the original global database [30] (not including observations in ref. [21]). Furthermore, in view of the potential underestimation of N_2_ fixation rates obtained using the original ^15^N_2_ gas bubble method [65,66], excluding these observations (alongside those measured by the acetylene reduction assay) produced much higher average rates of 142 (geometric) and 197 (arithmetic) μmol N m^−^^2^ d^−^^1^ (Table S6). Multiplying these N_2_ fixation rates by an area of 7.2 × 10^6^ km^2^ (area in the North Pacific highlighted with a red line in Fig. 3), resulted in a total N flux of 5.2 (geometric, or 7.2 for arithmetic) Tg N yr^−1^ (Table 2 and Table S6).

**Table 2.** Estimation of N_2_ fixation flux in the western North Pacific and three other potential UCYN-B dominated regions. Geometric estimates are in bold, and arithmetic estimates are shown in parentheses. The observed and three potential regions refer to red line highlighted areas in the western North Pacific, western South Pacific, Indian Ocean and eastern South Atlantic (Fig. 3). Since observational data are only available in the western North Pacific (Fig. S8a), the average N_2_ fixation rate (142 for geometric and 197 for arithmetic μmol N m^−2^ d^−1^, Table S6) measured using ^15^N_2_ dissolution method in this and previous studies [21,67,68] in the western North Pacific was used to estimate the flux in these regions.

Regions	Area (× 10^6^ km^2^)	Estimated flux based on 3° × 3° re-gridded datasets in the global database [30] (Tg N yr^−1^)	Re-evaluated flux based on N_2_ fixation rates measured in refs. [21,67,68] and this study (Tg N yr^−1^)	Additional flux (Tg N yr^−1^)
**Observed region**				
Western North Pacific	7.18	**4.3** (4.7)	**5.2** (7.2)	**0.9** (2.5)
**Potential regions**				
South Pacific	2.20	**2.1** (2.4)	**1.6** (2.2)	**−0.5** (−0.2)
Indian Ocean	5.10	**0.3** (0.3)	**3.7** (5.1)	**3.4** (4.8)
South Atlantic	0.45	**0.1** (0.1)	**0.3** (0.5)	**0.2** (0.4)
**Total predicted regions**	14.93	**6.8** (7.5)	**10.8** (15.0)	**4.0** (7.5)

In the other three UCYN-B-dominated, high N_2_ fixation rate areas (7.7 × 10^6^ km^2^) predicted by the GAMs (Fig. 3), the number of N_2_ fixation rate observations is very limited (Fig. S8). Using the average rate we measured in the western North Pacific, we estimated that collectively these three predicted UCYN-B dominated hot spots alongside the western North Pacific could potentially contribute a total of 10.8 (geometric, or 15.0 for arithmetic) Tg N yr^−^^1^ to the global marine N_2_ fixation budget (Table 2). The N_2_ fixation fluxes of these regions were also estimated based on 3° × 3° re-gridded datasets from the global database [30] as a comparison (Table 2). Our re-evaluated N_2_ fixation fluxes showed an additional N input of 4.0 (geometric, or 7.5 for arithmetic) Tg N yr^−1^, an ∼60%–100% increase from the estimate based on the global database [30]. The increase is more pronounced in the previously under-sampled regions, especially the western Indian Ocean which showed an additional N_2_ fixation flux of 3.4 (geometric, or 4.8 for arithmetic) Tg N yr^−1^, an increase of more than 10-fold (Table 2).

## CONCLUSIONS

N_2_ fixation by diazotrophic bacteria is a crucial source of biologically available N, supporting primary production in the global ocean [4]. Our study demonstrates that UCYN-B can dominate the diazotroph community and establish hot spots of N_2_ fixation, contributing significantly to global marine N_2_ fixation. Particularly, in the predicted UCYN-B dominated western Indian Ocean, a highly under sampled region, it may attribute an additional 45%–52% of N^2^ fixation for the whole Indian Ocean (7.5 geometric, or 9.2 Tg N yr^−1^ for arithmetic [69]). Future changes in environmental conditions will likely alter the spatial extent of the niches of the different diazotrophs, impacting both overall rates and fates of diazotroph fixed N [58], with knock-on impacts on ocean productivity and biogeochemical cycles [70]. Continued exploration of diazotrophs and N_2_ fixation throughout unsampled regions of the global ocean remains important in order to better understand and predict such changes.

## METHODS

### Study location and sample collection

Two GEOTRACES Process Study cruises were conducted aboard the R/V *Tan Kah Kee* from July to August 2020 and December 2020 to February 2021, respectively, in the (sub)tropical western North Pacific. Seawater samples were collected using a rosette of Niskin^®^ bottles equipped with SBE 911plus CTD (Seabird, Bellevue, USA) including a photosynthetically active radiation (PAR) sensor (Biospherical Instruments, San Diego, USA), which also recorded percentage light intensity relative to the surface and seawater temperature. Water samples were collected from six different depths (corresponding to 100%, 50%, 25%, 10%, 1% and 0.1% PAR) for N_2_ fixation rate incubations and for sampling for *nifH* gene quantification. Surface water was collected for *nifH* gene sequencing and metagenomics analysis. Information of sampling locations and types of analysis are listed in Table S1.

### N_2_ fixation rate measurement

N_2_ fixation rates were determined using the ^15^N_2_ gas dissolution method [66]. Prior to incubation, ^15^N_2_ pre-dissolved seawater was prepared using ^15^N_2_ gas (98.9 atom%, Cambridge Isotope Laboratories, Lot #: I-19 197/AR0586172, I-21 065/AR0664758). Specifically, seawater filtered through a 0.22-μm polypropylene calyx capsule filter (GVS S.p.A., Bologna, Italy) was collected around the observation sites and then degassed using a Sterapore membrane unit (20M1500A: Mitsubishi Rayon Co. Ltd., Tokyo, Japan). After this, 20 mL of ^15^N_2_ gas was injected into a Tedlar^®^ PVF gas-tight plastic bag (Delin Gas Packing Co., Ltd, Dalian, China) that contained 2 liters of the degassed seawater. The bag was gently tapped until the gas bubble was completely dissolved. The ^15^N_2_ pre-dissolved seawater was prepared at least 2 hours before initiation of the incubation. For bulk N_2_ fixation rate incubations, 100 mL ^15^N_2_-enriched seawater was added to duplicate 4.5-liter Nalgene polycarbonate bottles already filled with seawater directly from Niskin^®^ bottles. For size-fractionated (<10 μm) N_2_ fixation rate incubations, 60 mL ^15^N_2_-enriched seawater was added to a 2.3-liter bottle filled with seawater that was first filtered through a 10-μm membrane (TCTP04700 Isopore^TM^ hydrophilic membrane, Merck KGaA, Darmstadt, Germany) to remove large diazotrophs. All bottles were then incubated for 24 hours in on-deck incubators screened at six different levels of PAR (100%, 50%, 25%, 10%, 1% and 0.1% PAR) and flushed continuously with surface seawater. The upper euphotic zone depth-integrated N_2_ fixation rates were calculated by the trapezoidal integration method [71].

### Detection limits and error propagation

Particulate nitrogen (PN) concentrations and ^15^N isotopic ratios were quantified using an EA IsoLink^TM^ IRMS system (a Flash IRMS elemental analyzer coupled to a Delta V isotope ratio mass spectrometer, Thermo Scientific). N_2_ fixation rates were calculated as described by Montoya *et al.* [72]. Limits of detection (LOD) was calculated following the best practice described by White *et al.* [73] (Supplementary Data 1). Specifically, LOD-1 was derived from the minimal detectable difference in the ^15^N atom% of PN (^min^∆*A*_PN_) that can be resolved by the EA-IRMS given a sample PN mass. In our study, the PN masses of 82.8% of the bulk incubation samples were >10 μg, but the PN masses of most <10-µm-fraction incubation samples were in the range of 4–10 μg. For the PN mass <10 μg, the ^min^∆*A*_PN_ was three times the standard deviation of five *A*_PN_ measurements (0.00017 atom%) of low-mass N standards (1.52–3.52 μg), yielding a ^min^∆*A*_PN_ of 0.0005 atom% (*n* = 5, Table S7), which was lower than the generic value of 0.00146 atom% [72]. We thus used the 0.00146 atom% as the ^min^∆*A*_PN_ in the LOD-1 calculation for all samples. LOD-2 was also calculated by employing standard error propagation based on the variability observed between duplicate samples [73].

Measurement of ^15^N_2_ atom% was not conducted for the summer cruise (July to August 2020). For the winter cruise (December 2020 to February 2021), the samples for measurement of ^15^N_2_ atom% were collected into Exetainer^TM^ vials without a headspace, kept at 4°C, and then measured using a membrane inlet mass spectrometry (Prismaplus MIMS200, Bay Instruments, USA). The average value of ^15^N_2_ atom% for incubation of bulk N_2_ fixation rate measurement was 1.41 atom% with a low standard deviation of 0.083 atom% (*n* = 81). The average value for size-fractionated (<10 μm) incubation was 1.56 atom% with standard deviation of 0.077 atom% (*n* = 27). Given the consistent methodology applied in the summer and winter cruises, we used ^15^N_2_ enrichment values of 1.41 and 1.56 atom% in calculating bulk and size-fractionated N_2_ fixation rates, respectively. For the error propagation analysis, a fixed standard deviation of 0.083 atom% was used for bulk N_2_ fixation rate measurement. Error propagation analysis was not performed for size-fractionated N_2_ fixation rate measurements, due to lack of replicates as a result of limited seawater samples available for incubation on the cruises. Data are shown in Supplementary Data 1.

### *nifH* gene sequencing and quantification

A total of 4.5L *in-situ* seawater samples were filtered onto 0.22 μm pore-sized polyethersulfone (PES) membranes (Supor-200, Pall Gelman), which were subsequently frozen in liquid N_2_ until further analysis. To extract DNA, membranes were cut into pieces under sterile conditions and then extracted using the QIAamp® DNA Mini Kit (Qiagen) following the manufacturer's protocol with minor modification by adding 20 μL lysozyme (20 mg/mL) for cell lysis. The concentration and purity of the genomic DNA were detected using a Qubit Fluorometer (Qubit^TM^ 4 Invitrogen) and a NanoDrop spectrophotometer (Thermo Scientific 2000/2000c), respectively.

Surface sea water samples from six stations in summer and four stations in winter were collected for *nifH* gene DNA sequencing. Among them, four stations in summer (K2b, WPS, MR04 and M22) and two stations in winter (M22_W and K11a_W) had high N_2_ fixation rates. Two stations in summer (K11a and K8a) and two stations in winter (MR04_W and K8a_W) that had very low or no detectable N_2_ fixation rates were included as comparison (see Table S1 for station information). *nifH* gene amplification was performed using previously reported nested PCR protocols [74,75]. Purified amplicons were pooled in equimolar amounts and paired-end sequenced on an Illumina MiSeq PE300 platform (Illumina, San Diego, USA) according to the standard protocols by Majorbio Bio-Pharm Technology Co. Ltd. (Shanghai, China). Sequence reads were demultiplexed using an in-house perl script, quality-filtered by fastp version 0.19.6 [76] and then merged by FLASH version 1.2.7 [77]. The optimized sequences were then clustered into operational taxonomic units (OTUs) using UPARSE 7.1 [78,79] at 97% nucleotide similarity. The most abundant sequence of each OTU was selected as the representative sequence. The taxonomy of the representative sequences was identified by RDP Classifier version 2.2 [80] against the National Center for Biotechnology Information (NCBI) nucleotide database.

Quantitative polymerase chain reaction (qPCR) analysis was applied to samples collected from six different depths (corresponding to 100%, 50%, 25%, 10%, 1% and 0.1% PAR) for 26 stations (Table S1). Five prominent *nifH* phylotypes including *Trichodesmium*, UCYN-A1, UCYN-A2/A3, UCYN-B and *Richelia* associated with *Rhizosolenia* (Het-1), which were identified by *nifH* amplicon sequencing, were quantified using previously designed primers and probe sets (Table S8). Triplicate qPCR reactions were run for each DNA sample on a CFX96 Real-Time System (Bio-Rad Laboratories). A set of standards corresponding to between 10^0^ and 10^6^ copies per well were amplified in each 96-well plate. The qPCR efficiencies were between 93.3%–108.8%, with *R*^2^ value >0.99. The detection limit was 25 *nifH* copies per liter and the quantification limit was 250 *nifH* copies per liter. No signals were detected in negative controls. The *nifH* gene standards were obtained by cloning the environmental sequences from previous samples collected from the South China Sea.

### Metagenomic analysis

Surface seawater samples were collected for metagenomics analysis at stations K2b, M22, WPS, K8a and M35 (see Table S1 for station information). M22, WPS and K2b all had high N_2_ fixation rates, while a *Trichodesmium* bloom occurred at K2b when sampling. K8a had no detectable N_2_ fixation rate and was included as a comparison. M35 located at the higher latitude and had a low N_2_ fixation rate. Seawaters were sampled at two size fractions, i.e. 0.2–3 μm and 3–200 μm. DNA was extracted as described above, and the metagenomics libraries were prepared using an Automation System (MGI, MGISP-960, China) with MGIEasy Universal DNA Library Prep Set (MGI 1000006 986, China), using an input of 1 μg of sheared genomic DNA. Non-redundant *nifH* genes were constructed, and then annotated for downstream analysis (see Note S4 for detailed description).

## Supplementary Material

nwaf337_Supplemental_Files

## Data Availability

All data are available in the main text or the Supplementary Materials. The fastq files of *nifH* sequencing in this study have been deposited in NCBI (Accession: PRJNA992762). All other source data are provided in the Supplementary Data. Correspondence and requests for data should be addressed to the corresponding author.
