# Supplementary material for: Significant contribution of the unicellular cyanobacterium UCYN-B to oceanic nitrogen fixation
Source: Natl Sci Rev. 2025 Aug 14;12(10):nwaf337. doi: 10.1093/nsr/nwaf337 (PMC12485613; doi:10.1093/nsr/nwaf337)
Supplement: nwaf337_Supplemental_Files [file nwaf337_supplemental_files.zip › Jiang et al UCYN-B Supplementary Information.pdf]

Supplementary Information

The Supplementary file includes:

Supplementary Note 1–4;

Supplementary Table 1–9;

Supplementary Figure 1–8;

Supplementary Data List;

Supplementary References.

Supplementary Note 1. Estimation of UCYN-B derived N_2 fixation rates based on UCYN-B cell-specific rate, polyploidy factor and *nifH* abundance.

Cell-specific N_2 fixation rates of UCYN-B measured using nanometer-scale secondary ion mass spectrometry (nanoSIMS) technology were compiled [42–44], which showed an average rate of 12.4 fmol N $cell^{-1}$ d^{-1} (Supplementary Table 3). Recent studies have evidenced the existence of *nifH* gene polyploidy in various diazotrophs including UCYN-B[37]. We thus converted the cell-specific rate into *nifH*-specific rate using an average polyploidy factor of 3.6 [26,45] for UCYN-B, resulting in an average *nifH*-specific rate of 3.43 fmol N $nifH^{-1}$ d^{-1} of UCYN-B (Supplementary Table 3, Eq. S1). Then, the UCYN-B derived N_2 fixation rates at each station were calculated based on the measured UCYN-B *nifH* abundance and *nifH*-specific rate (Eq. S2), from which a contribution of UCYN-B to measured bulk N_2 fixation rate was estimated at each station with detectable N_2 fixation rate (Supplementary Table 4)

nifH-specific N_2 fixation rate = cell-specific N_2 fixation rate ／average polyploidy factor (Eq. S1)

UCYN-B derived N_2 fixation rate = *nifH*-specific rate × UCYN-B *nifH* gene abundance (Eq. S2)

Supplementary Note 2. GAM analysis

Depth-integrated *nifH* gene abundances were obtained from the global database summarized by ref. [30] and the additional observations in this study. We focus on four major diazotroph phylotypes in the global ocean, *Trichodesmium*, UCYN-A, UCYN-B

and *Richelia*, and in total 737 data points of each phylotype were included in the following Generalized Additive Model (GAM) analysis (Supplementary Figure 5).

The ecological niches of diazotrophs were characterized based on the responses of the diazotroph groups to key environmental variables using GAM [81,82]. The 'mgcv' package for GAM is available online (https://CRAN.Rproject.org/package=mgcv). Various physical and chemical factors including temperature, light intensity and nutrient concentrations (i.e., dissolved Fe, phosphate and nitrate) regulate the distribution and activity of diazotrophs in the ocean [83]. Specifically, 1) Most enzyme-mediated reactions, including N_2 fixation, are regulated by temperature [84]; 2) For photoautotrophic diazotrophs, nitrogenase activity is intimately linked to photosynthesis [85], and thus controlled by light intensity; 3) Fe is an essential cofactor for the nitrogenase enzyme [86]; 4) Phosphorus is essential for information storage (DNA and RNA) and energy generation [adenosine 5′-triphosphate (ATP)] molecules; 5) Nitrate is not necessarily required by diazotrophs (having the ability to fix N themselves); however, diazotrophs may be outcompeted by faster growing non-diazotrophs in regions with elevated N supply [21,58].

As these five ancillary data types were not available for many of the observations of *nifH* gene abundance in the dataset, we performed the GAMs analysis using model simulated monthly climatologies of these environmental variables [sea surface temperature (SST),

surface dissolved Fe (dFe), phosphate (P) and nitrate (N), and photosynthetically active radiation (PAR)], to keep the consistency of data sources. SST, dFe, P and nitrate (N) were downloaded from CMIP6-CESM2 (https://pcmdi.llnl.gov/CMIP6/) [87]. PAR was derived from the Community Earth System Model Large Ensemble Numerical Simulation (CESM-LENS) project (https://www.cesm.ucar.edu/community-projects/lens) [88]. The PAR had strong spatial correlation ($R = 0.63$, $P < 0.05$) with SST, thus distinguishing between the two environmental factors is not necessary for the global mapping of diazotrophs [63]. In addition, single-variable GAM analysis showed lower explanatory power of PAR to *nifH* gene abundance compared to other variables, especially for *Trichodesmium* and UCYN-B (Supplementary Table 9). Therefore, PAR was excluded from the GAMs analysis. In order to improve the model performance, we hereafter conducted a stepwise addition analysis by adding the other three variables (dFe, P and N) into the SST GAM (Supplementary Table 5). The addition of nutrients either alone or simultaneously significantly improved the model's explanatory power to all diazotrophs. However, we also found that the four-variable GAM (SST + dFe + P + N) did not improve the explanatory powers significantly compare to that of adding dFe+P and dFe + N. In addition, *Trichodesmium* did not appear to correlate with all variables in the four-variable-GAM ($P > 0.05$). It is thus more reliable to use a three-variable GAM. In this study, the diazotroph niches were estimated using the SST + dFe + P GAM which performed best in explaining the variations of diazotroph abundances (Supplementary Table 5).

Supplementary Note 3. Global projection of diazotroph distribution

10-year averaged, monthly-resolution SST, dFe and P from CMIP6-CESM2 were then used to predict diazotroph abundances based on the relationships between environmental variables and *nifH* gene abundances produced by the SST + dFe + P GAMs (Figure 2 and Supplementary Figure 6). Our predictions were restricted to oceanic regions between 40ºS and 45ºN with SST above 15°C, as most of the *nifH* gene abundance data used in the GAMs were obtained from these regions (Supplementary Figure 5).

Supplementary Note 4. Metagenomics analysis

Samples were sequenced on the DNBseq platform (Beijing Genomics Institute, Shenzhen), and the raw paired-end short reads (2 × 150 nt) were filtered with SOAPnuke [89]. 0.4 billion quality filtered reads from two size fraction samples in each station were co-assembled using MEGAHIT (v1.2.9) [90], with a minimum contig length of 1,000 bp. Open reading frames within the contigs were identified using Prodigal (v2.6.3) [91]. Then we used the eggNOG-mapper (v2.0) [92] to search the eggNOG database [93] to infer and assign the functions to genes in our contigs. All complete *nifH* sequences were clustered at a 95% protein identity level after functional annotation using cd-hit (v4.8.1) [94]. A *nifH* sequence catalog was constructed, which were composed of all *nifH* sequences extracted from our samples above. Moreover, this catalog was complemented with additional *nifH* sequences retrieved from another diazotrophic genomic database

[48]. *NifH* protein sequences in the catalog were aligned to the Uniprot TrEMBL database using DIAMOND (v2.0.14) [95] with an e-value ≤ $1e^{-5}$. Taxonomic classification in the catalog was annotated through the lowest common ancestor algorithm by BASTA (v1.4) [96] at the thresholds of an alignment length > 25, identity > 80%, and shared by at least 60% of hits for matching distinct taxonomic groups. Quality filtered reads in our samples were mapped to the *nifH* catalog using Bowtie 2 (v2.4.1) [97] with default settings and the additional '-no-unal' flag. SAM files were then sorted and indexed using Samtools (v1.7) [98] and Sambamba (v0.8.1) [99], and the transcripts per kilobase million (TPM) values were calculated with coverM (v0.6.1, https://github.com/wwood/CoverM).

Supplementary Table 1. Information of sampling stations and different types of analysis. Two cruises were conducted in the (sub)tropical western North Pacific in summer (17 stations) and winter (10 stations), respectively. The study area was simply divided into three regions, including the center area of the North Pacific Subtropical Gyre (NPSG), the North Pacific Transition Zone (NPTZ), and the North Equatorial Current affected area (NEC).

Cruise	Station	Area	Bulk depth-integrated NFR (µmol N m^{-2} d^{-1})[a]	Size-fractionated NFR[a, b]	*nifH* qPCR[a]	*nifH* sequencing[c]	Metagenomic analysis[d]
Summer	K2b[e]	NPSG	198.7	N/A	√	√	√
	KPR	NPSG	758.9	N/A	N/A	N/A	N/A
	WPS	NPSG	787.9	N/A	√	√	√
	MR04	NPSG	585.7	√	√	√	N/A
	M22	NPSG	820.9	√	√	√	√
	M20	NPSG	289.5	N/A	√	N/A	N/A
	M18	NPSG	711.1	√	√	N/A	N/A
	MR05	NPTZ	17.8	N/A	√	N/A	N/A
	M35	NPTZ	62.6	√	√	N/A	√
	M32	NPTZ	41.8	N/A	√	N/A	N/A
	M30	NPTZ	100.2	√	√	N/A	N/A
	M26a	NPTZ	78.6	√	√	N/A	N/A
	M16	NEC	76.2	N/A	√	N/A	N/A
	K8a	NEC	Not Detected	√	√	√	√
	K11a[f]	NEC	Not Detected	N/A	√	√	N/A
	K12a	NEC	85.1	N/A	√	N/A	N/A
	K13	NEC	6.5	N/A	√	N/A	N/A
Winter	MR04_W	NPSG	20.4	√	√	√	N/A
	M22_W	NPSG	158.0	√	√	√	N/A
	M18_W	NPSG	Not Detected	√	√	N/A	N/A
	K11a_W[f]	NPSG	228.0	N/A	√	√	N/A
	MR05_W	NPTZ	8.9	√	√	N/A	N/A
	M30_W	NPTZ	218.4	√	√	N/A	N/A
	K8a_W	NEC	Not Detected	√	√	√	N/A

K9a_W	NEC	Not Detected	N/A	√	N/A	N/A
K12a_W	NEC	Not Detected	√	√	N/A	N/A
K13a_W	NEC	90.7	√	√	N/A	N/A

N/A: not sampled or measured

[a] Water samples were collected from six different depths (corresponding to 100%, 50%, 25%, 10%, 1% and 0.1% PAR) for N_2 fixation rate measurement and *nifH* gene qPCR.

[b] Profile bulk N_2 fixation rates were determined at all of stations, but size-fractionated (< 10 μm) N_2 fixation rates were determined at 7 stations in summer and 8 stations in winter due to the sample limitation during cruises.

[c] Surface sea water samples from 6 stations in summer and 4 stations in winter were collected for *nifH* DNA sequencing. Among them, four stations in summer (K2b, WPS, MR04 and M22) and two stations in winter (M22_W and K11a_W) had high N_2 fixation rates. Two stations in summer (K11a and K8a) and two stations in winter (MR04_W and K8a_W) that had very low or no detectable N_2 fixation rates were included as comparison.

[d] Surface sea water samples from three stations with high N_2 fixation rates (K2b, WPS, M22), one station with low N_2 fixation rates (M35) and one station with no detectable rates (K8a) were collected for metagenomic sequencing.

[e] A *Trichodesmium* bloom occurred at station K2b in summer.

[f] Station K11a was affected by NEC in summer but not in winter based on characteristics of surface water and NEC position analysis.

Supplementary Table 2. Diazotroph community structure in the surface waters of western North Pacific in summer based on metagenomics analysis. M22, WPS and K2b were stations with high N_2 fixation rate (> 100 μmol N m^{-2} d^{-1}). The dominant diazotroph was UCYN-B at M22 and WPS in both of the two size fractions. A *Trichodesmium* bloom occurred at K2b when sampling and *Trichodesmium* indeed was found to be most abundant in the size fraction of 3–200 μm, while UCYN-B was still dominant in the fraction of 0.2–3 μm. K8a had no detectable N_2 fixation rate and was included as a comparison, where non-cyanobacterial diazotrophs (NCDs) were dominant. M35 was a low N_2 fixation rate station at higher latitudinal North Pacific Transition Zone (NPTZ). UCYN-A was the most abundant cyanobacterial diazotroph in M35, while the most abundant diazotrophs were NCDs. The TPM table and taxonomy for all *nifH* sequences are provided in Supplementary Data 6.

Station	Size fraction (μm)	Cyanobacterial diazotrophs				Non-cyanobacterial diazotrophs (NCDs)			Others
		Trichodemsium	**UCYN-B**	**UCYN-A**	***Richelia***	**Gamma**	**Alpha**	**Other NCDs**	
M22	0.2–3.0	0.0%	64.5%	0.0%	0.0%	7.2%	23.8%	4.4%	0.0%
	3.0–200	1.6%	78.1%	0.0%	0.8%	1.9%	15.7%	1.4%	0.6%
WPS	0.2–3.0	0.0%	81.4%	0.0%	0.0%	1.3%	11.3%	4.8%	1.2%
	3.0–200	12.9%	74.0%	0.0%	0.4%	2.7%	8.8%	0.7%	0.6%
K2b	0.2–3.0	1.1%	72.7%	0.0%	0.0%	7.4%	10.9%	6.4%	1.5%
	3.0–200	89.2%	9.6%	0.0%	0.2%	0.3%	0.4%	0.0%	0.3%
K8a	0.2–3.0	0.0%	2.7%	0.0%	0.0%	12.0%	78.2%	6.8%	0.0%
	3.0–200	2.0%	5.8%	0.0%	0.6%	12.3%	74.2%	5.1%	0.0%
M35	0.2–3.0	0.0%	0.0%	19.0%	0.0%	31.0%	45.7%	4.4%	0.0%
	3.0–200	0.0%	0.0%	2.1%	0.0%	2.8%	80.8%	13.5%	0.7%

Supplementary Table 3. UCYN-B cell-specific and *nifH*-specific N_2 fixation rates.

The cell specific N_2 fixation rates were measured by nanoSIMS [42–44] and the *nifH*-specific N_2 fixation rates were calculated by dividing cell specific rate with the polyploidy factor of 3.6* [26,45].

**Cell-specific UCYN-B N_2 fixation rates (fmol N cell^{-1} d^{-1})**	**UCYN-B *nifH*-specific N_2 fixation rate (Cell specific rate/3.6, fmol N *nifH*$^{-1}$ d^{-1}) ***	**Experimental location and field incubation time**	**Data Source**
30.0	8.33	South Pacific, 24 h of field incubation	[42]
6.1	1.69	South Pacific, 24 h of field incubation	
4.2	1.15	North Atlantic, 13.2 h of field incubation	[43]
13.4	3.73	North Atlantic, 23.0 h of field incubation	
3.4	0.93	ALOHA, 3.8 h of field incubation, single cells	[44]
12.0	3.33	ALOHA, 12.3 h of field incubation, single cells	
18.2	5.07	ALOHA, 18.3 h of field incubation, single cells	
6.2	1.73	ALOHA, 3.8 h of field incubation, colonial cells	
14.2	3.93	ALOHA, 12.3 h of field incubation, colonial cells	
15.8	4.40	ALOHA, 18.3 h of field incubation, colonial cells	
Average			
12.4 ± 7.7	3.43 ± 2.13		

*Average polyploidy factor of 3.6 for UCYN-B was cited from Ref. [45] and used for conversion from cell specific rates to *nifH* specific rates. The polyploidy factor varied in the range of 2.5–5.9 based on the data from Ref. [26].

Supplementary Table 4. Estimated contribution of UCYN-B to measured bulk N_2 fixation rate in western North Pacific using literature values of cell-specific N_2 fixation rates measured by nanometer-scale secondary ion mass spectrometry (nanoSIMS). High contribution of UCYN-B to N_2 fixation rate (> 50%) was found at stations in North Pacific Subtropical Gyre (NPSG) and North Equatorial Current (NEC) affected regions where high UCYN-B *nifH* gene abundance and high proportion in diazotrophs community was observed (yellow background). A *Trichodesmium* bloom occurred at Station K2b although it was classified as high rate NPSG station (green background). UCYN-B contributed non-significantly to stations in North Pacific Transition Zone (NPTZ) as well as MR04_W (grey background). UCYN-B contribution was not estimated for stations with no detectable N_2 fixation rates.

**Station**	**Depth-integrated NFR (μmol N m^{-2} d^{-1})**	**UCYN-B *nifH* gene abundance (copies m^{-2})**	**Estimated UCYN-B NFR (μmol N m^{-2} d^{-1})[a]**	**UCYN-B *nifH* gene proportion[b]**	**Estimated UCYN-B contribution to bulk NFR**	**Note**
K2b	198.7	1.94E+10	66.6	39.9%	33%	NPSG, *Trichodesmium* bloom
WPS	787.9	1.71E+11	586.7	98.8%	74%	NPSG
MR04	585.7	7.84E+10	269.0	86.7%	46%	NPSG
M22	820.9	2.22E+11	761.7	100.0%	92%	NPSG
M20	289.5	8.24E+10	282.7	99.9%	97%	NPSG
M18	711.1	9.81E+10	336.6	99.9%	47%	NPSG
M22_W	158.0	4.95E+10	169.8	99.8%	106 %	NPSG
K11a_W	228.0	6.84E+10	234.7	99.4%	102%	NPSG
K13a_W	90.7	3.10E+10	106.4	98.7%	116%	NEC
K12a	85.1	3.25E+10	111.5	100.0%	130%	NEC
M16	76.2	6.46E+10	221.6	98.3%	288%	NEC
K13	6.5	3.20E+09	11.0	99.7%	166%	NEC

MR05	17.8	3.52E+09	12.1	14.7%	67%	NPTZ
M35	62.6	4.70E+08	1.6	1.2%	2.6%	NPTZ
M32	41.8	7.91E+07	0.3	1.0%	0.6%	NPTZ
M30	100.2	3.12E+08	1.1	1.0%	1.1%	NPTZ
M26a	78.6	7.18E+07	0.2	3.0%	0.3%	NPTZ
MR05_W	8.9	8.19E+07	0.3	8.3%	3.1%	NPTZ
M30_W	218.4	1.27E+08	0.4	0.9%	0.2%	NPTZ
MR04_W	20.4	3.17E+07	0.1	9.2%	0.5%	NPSG

[a] UCYN-B derived N_2 fixation rate was estimated based on the cell-specific N_2 fixation rates measured using nanometer-scale secondary ion mass spectrometry (nanoSIMS) [42–44], the number of *nifH* gene polyploidy [26,45] and the depth-integrated UCYN-B *nifH* abundance (detail description in Supplementary Note 1).

[b] UCYN-B *nifH* gene proportion was calculated by dividing the depth-integrated UCYN-B *nifH* abundance by the total *nifH* abundance of five cyanobacterial diazotrophs (Figure 1b).

Supplementary Table 5. Stepwise GAM analysis using environmental variables including SST, dFe, P and N. R-sq represents the determination coefficient of the GAMs that produced relationship between *nifH* gene abundances and environmental variables. Dev-exp is the deviance explanation of variable sets to the variation of *nifH* abundances. Our new observations in this study were incorporated into a global database of depth-integrated *nifH* gene abundances [30] to conduct GAMs analysis (Supplementary Figure 5, Supplementary Note 2).

**GAMs**	***Trichodesmium*** (n = 652)		**UCYN-B** (n = 605)		**UCYN-A** (n = 633)		***Richelia*** (n = 527)	
	R-sq	Dev-exp	R-sq	Dev-exp	R-sq	Dev-exp	R-sq	Dev-exp
SST + dFe	0.425	**43.30%**	0.433	**44.00%**	0.285	**29.30%**	0.331	**34.10%**
SST + dFe + P	0.508	**52.20%**	0.539	**54.90%**	0.401	**41.50%**	0.427	**44.00%**
SST + dFe + N	0.482	**49.40%**	0.519	**52.80%**	0.448	**45.80%**	0.414	**42.70%**
SST + dFe + P + N			0.548	**55.70%**	0.413	**42.30%**	0.423	**43.40%**

Supplementary Table 6. Estimation of N_2 fixation flux in the UCYN-B dominated western North Pacific. Geometric estimates are in bold, and arithmetic estimates are shown in parentheses. The predicted region in the western North Pacific refers to red line highlighted areas in the western North Pacific Gyre (Figure 3 and Supplementary Figure 8). The number of observations is different for geometric and arithmetic because zero-value data are excluded in calculating geometric means [64]. N_2 fixation rates in refs. [21,67,68] and this study are all measured using $^{15}N_2$ dissolution method.

**Regions**	**Area ($\times 10^6$ km^2)**	**Number of observations**	**N_2 fixation rate (μmol N m^{-2} d^{-1})**	**Estimated Flux (Tg N yr^{-1})**	**N_2 fixation rate data sources**
Western North Pacific	7.18	**49** (49)	**87** (177)	**3.2** (6.5)	refs. [30], excluding data in ref. [21]
		**85** (96)	**112** (192)	**4.1** (7.1)	refs. [30,67] and this study
		**41** (52)	**142** (197)	**5.2** (7.2)	refs. [21,67,68]and this study

Supplementary Table 7. Repeated measurements of N isotopic standards at the low mass (PN < 4 μg).

**Sample description**	**PN (μg)**	**$\delta^{15}N$ value (‰)**	**A_{PN} (Atom%)**
USGS40_low-mass (n =5)	1.523–3.522	-4.59 ± 0.45	0.3636% ± 0.00017%
ΔA_{PN} = 3 × standard deviation of A_{PN}			0.00050%

Supplementary Table 8. ***NifH*** **gene primers and probes used in quantitative PCR analyses.**

Species/clone	Forward primer	Probe	Reverse primer	Standard clone	References
Trichodesmium	GACGAAGTATTGAAGCCAGGTTTC	CATTAAGTGTGTTGAATCTGGTGGTCCTGAGC	CGGCCAGCGCAACCTA	AY528677	[100]
UCYN-A1	AGCTATAACAACGTTTTATGCGTTGA	TCCGGTGGTCCTGAGCCTGGA	ACCACGACCAGCACATCCA	AF059642	[100]
UCYN-A2/A3	GGTTACAACAACGTTTTATGTGTTGA	TCTGGTGGTCCTGAGCCCGGA	ACCACGACCAGCACATCCA	KF806604	[101]
UCYN-B	TGGTCCTGAGCCTGGAGTTG	TGTGCTGGTCGTGGTAT	TCTTCTAGGAAGTTGATGGAGGTGAT	AF299418	[100]
Het-1	CGGTTTCCGTGGTGTACGTT	TCCGGTGGTCCTGAGCCTGGTGT	AATACCACGACCCGCACAAC	AY706898	[102]

Supplementary Table 9. Deviance explanations and R^2 of single factor GAMs using five environmental parameters including SST, PAR, dFe, P and N.

Diazotrophs	*n*	SST		PAR		dFe		P		N	
		Dev-exp	R^2	Dev-exp	R^2	Dev-exp	R^2	Dev-exp	R^2	Dev-exp	R^2
Trichodesmium	652	**27.4%**	0.270	**4.38%**	0.038	**12.8%**	0.123	**35.7%**	0.353	**26.6%**	0.263
UCYN-B	605	**24.1%**	0.237	**3.35%**	0.027	**14.6%**	0.140	**38.1%**	0.377	**33.4%**	0.330
UCYN-A	633	**18.9%**	0.184	**5.12%**	0.045	**22.90%**	0.224	**10.9%**	0.103	**23.1%**	0.226
Richelia	527	**17.9%**	0.173	**5.32%**	0.046	**15.6%**	0.15	**33.7%**	0.332	**21.4%**	0.209

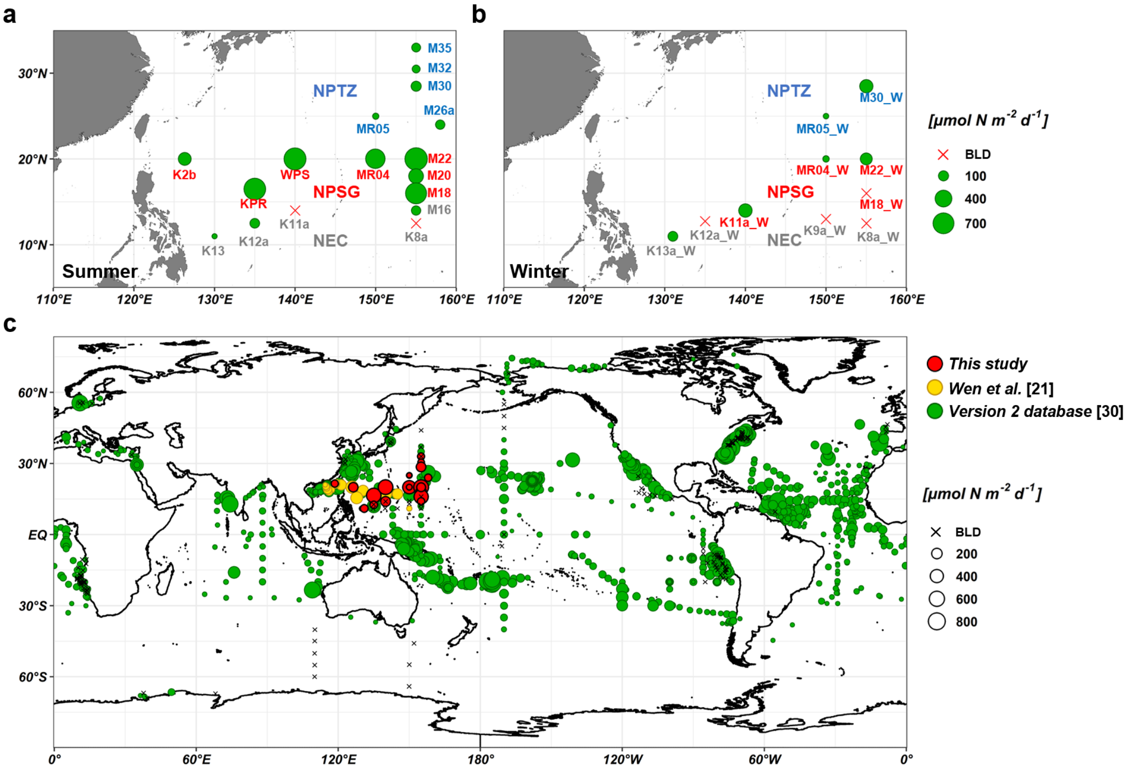

Supplementary Figure 1. Spatial distribution of depth-integrated N_2 fixation rates. Observations of N_2 fixation rates in (**a**) summer and (**b**) winter conducted in this study. The study area was simply divided into three regions, the center area of the North Pacific Subtropical Gyre (NPSG), the North Pacific Transition Zone (NPTZ), and the North Equatorial Current affected area (NEC). (**c**) The global distribution of N_2 fixation rates. The red and yellow dots in (**c**) represent our observations by this study and the previous study [21], respectively. Other data were from the global N_2 fixation database [30].

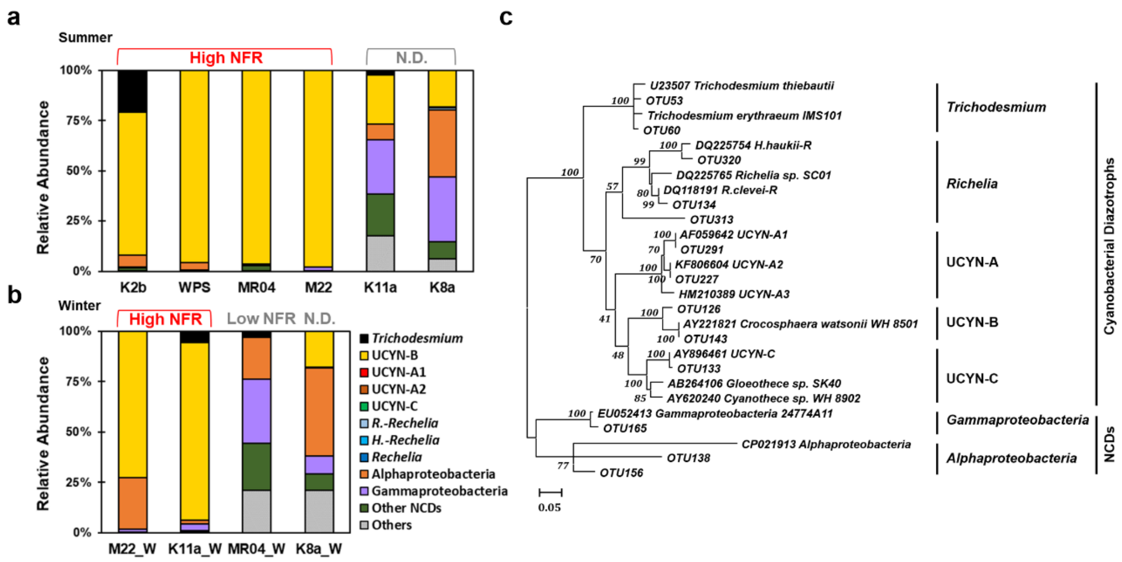

Supplementary Figure 2. Composition of the diazotroph community in surface waters of the western North Pacific stations in summer (a), winter (b) based on *nifH* gene DNA sequencing analysis, and maximum likelihood phylogenetic tree of *nifH* gene sequences (c). Station K2b, WPS, MR04, M22, M22_W and K11a_W were stations with high N_2 fixation rates (NFR, Supplementary Table 1). Station MR04_W was the station with low N_2 fixation. N_2 fixation rate was not detectable (N.D.) at station K11a, K8a and K8a_W (Supplementary Table 1). The 13 most abundant operational taxonomic units (OTU) accounted for > 95% of the reads recovered at high N_2 fixation rates stations in this study. Bootstrap values were determined from 1,000 iterations. OTU numbers and taxonomy for all sequences are provided in Supplementary Data 3–5.

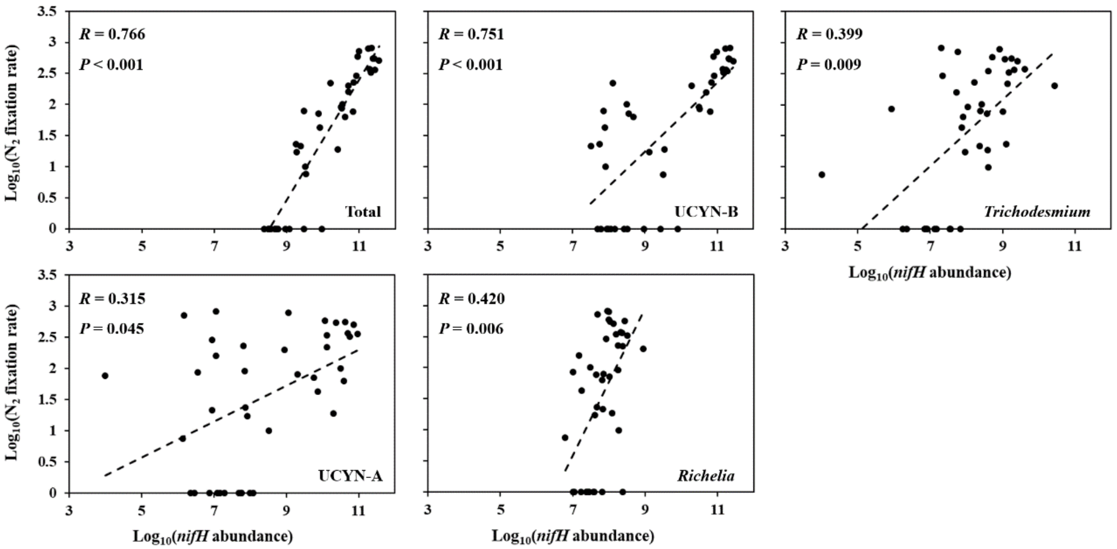

Supplementary Figure 3. Simple linear correlations (*n* = 40) between depth-integrated N_2 fixation rates and *nifH* gene abundances of four cyanobacterial diazotrophs. The observations in western North Pacific which included this study and Wen et al. [21] were compiled for analysis.

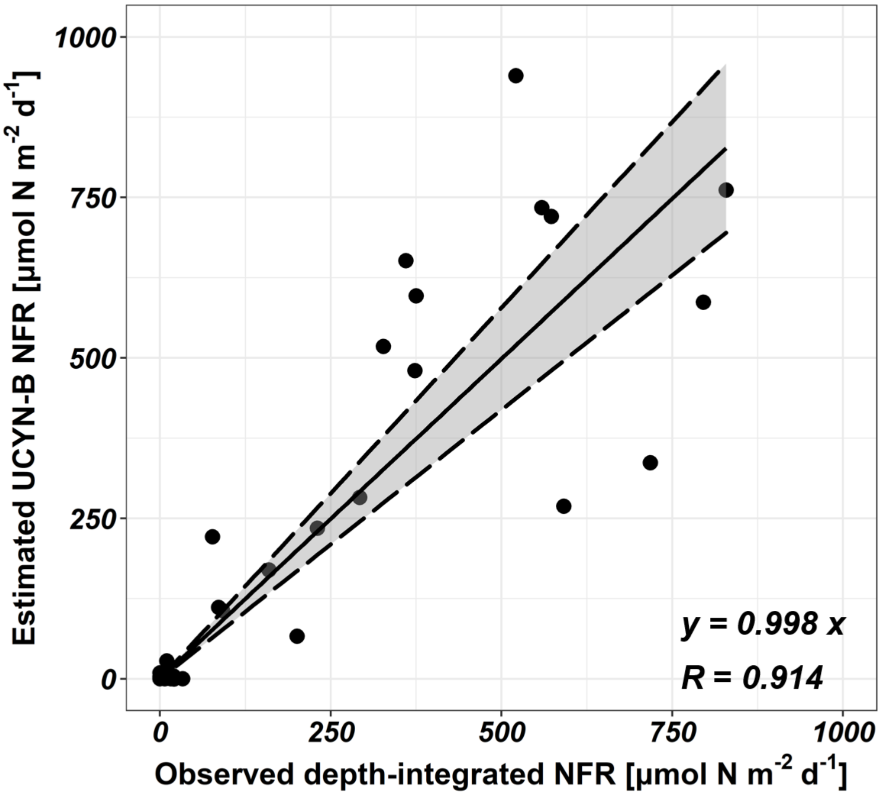

Supplementary Figure 4. Linear regression between estimated UCYN-B derived N_2 fixation rates and observed depth-integrated bulk N_2 fixation rates. UCYN-B derived N_2 fixation rates were estimated based on the cell-specific N_2 fixation rates measured using nanometer-scale secondary ion mass spectrometry (nanoSIMS) [42–44], the number of *nifH* gene polyploidy [26,45] and the depth-integrated UCYN-B *nifH* abundance (detail description in Supplementary Note 1). The observed N_2 fixation rates in NPSG and NEC from in this study and Wen et al. [21] were compiled for analysis.

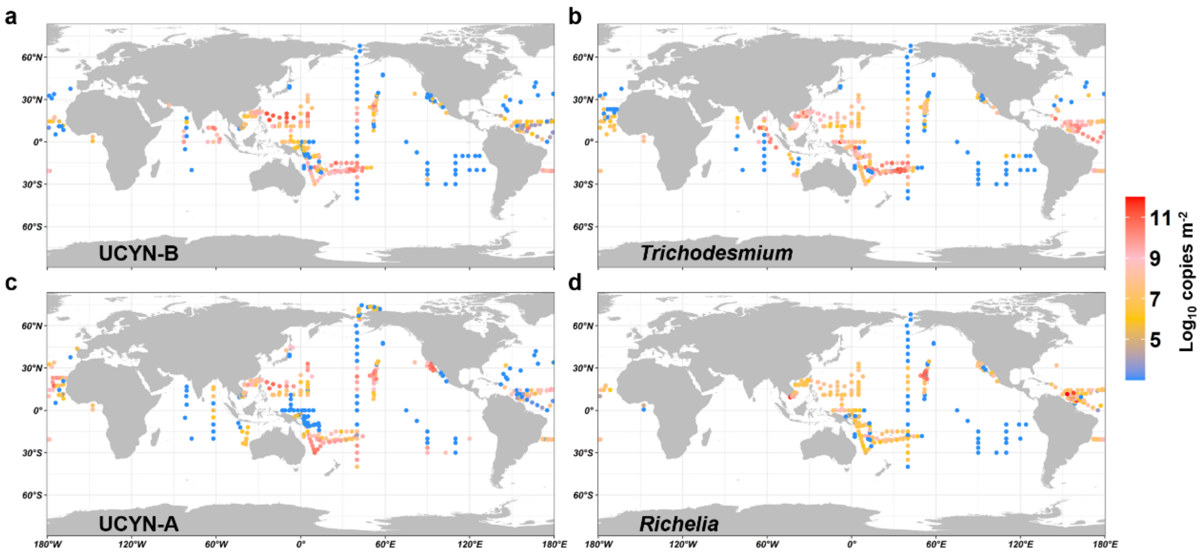

Supplementary Figure 5. Global distributions of the depth-integrated *nifH* gene abundance of four major diazotrophs. (**a**) UCYN-B, (**b**) *Trichodesmium*, (**c**) UCYN-A, and (**d**) *Richelia*. Apart from data in our study, all other data were obtained from the new global database of depth-integrated *nifH* gene abundance [30].

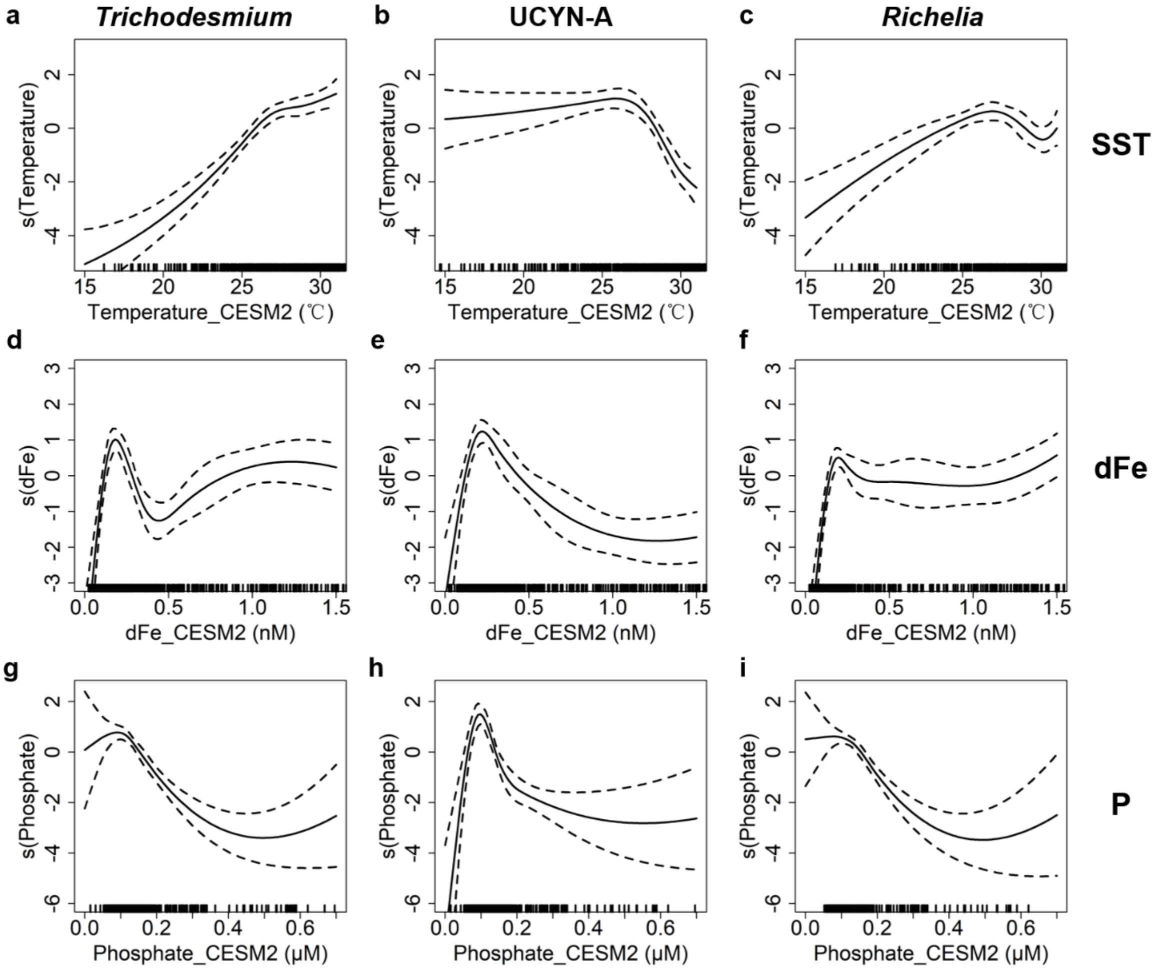

Supplementary Figure 6. GAM-modeled response of diazotroph abundance of *Trichodesmium*, UCYN-A and *Richelia* to (a-c) surface temperature (SST), (d-f) dissolved Fe (dFe), and (g-i) phosphate (P) concentrations. All diazotrophs correlated well with the three variables in the three-variable-GAM ($P < 0.05$). Our new observations in this study were incorporated into a global database of depth-integrated *nifH* gene abundances [30], to conduct GAMs analysis (Supplementary Figure 5, Supplementary Note 2).

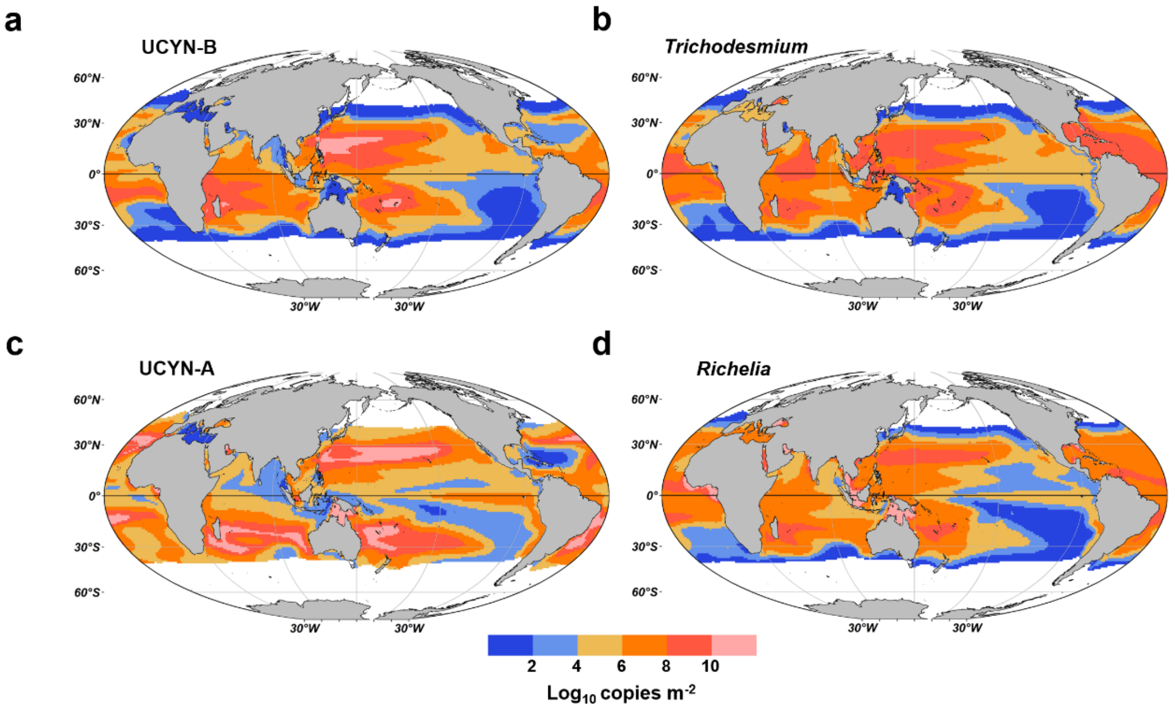

Supplementary Figure 7. Predicted global distributions of depth-integrated *nifH* abundance of four major diazotrophs. (**a**) UCYN-B, (**b**) *Trichodesmium*, (**c**) UCYN-A, and (**d**) *Richelia*. The prediction was performed by using the relationships revealed by the SST + dFe + P GAM (Figure 2a-c and Supplementary Figure 6) and 10-year averaged data of monthly SST, dFe and P from an ocean biogeochemical model simulation (CMIP6-CESM2).

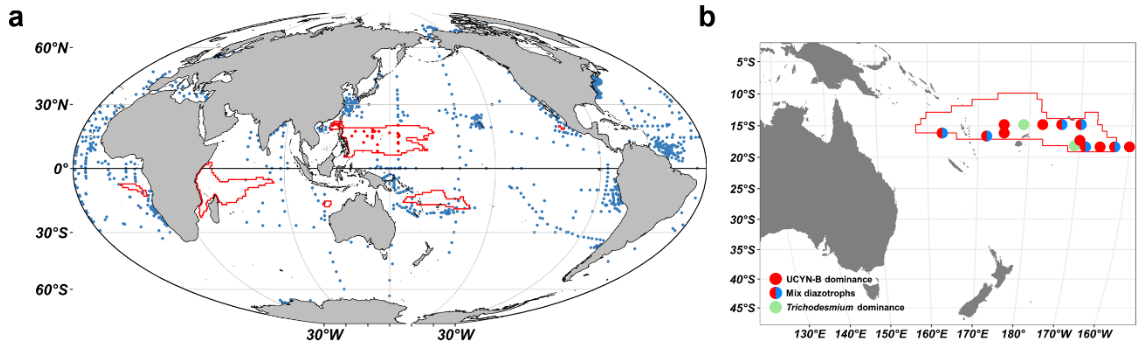

Supplementary Figure 8. Spatial distribution of depth-integrated data points in the N_2 fixation database. (**a**) Depth-integrated N_2 fixation rates data points in the database. The red line enclosed regions are predicted UCYN-B dominated areas with total *nifH* gene abundance higher than 10^8 copies m^{-2} and *nifH* gene abundance of UCYN-B > 75% of the total *nifH* gene (Figure 3). The dots are the station locations in the global database, and the red dots represent the observed stations located in our projected UCYN-B dominated area in the western North Pacific. (**b**) Dominating diazotrophs in the projected UCYN-B dominated areas in western South Pacific based on the *nifH* gene abundance reported in the database [30].

Supplementary Data. Source data for N_2 fixation, diazotrophs abundances *nifH* gene sequencing, and metagenomics analysis.

Supplementary Data 1: Profile dataset of bulk N_2 fixation rates

Supplementary Data 2: Depth-integrated datasets of N_2 fixation rates, diazotrophs abundances.

Supplementary Data 3: The optimized *nifH* sequences clustered into operational taxonomic units (OTUs) at 97% nucleotide similarity.

Supplementary Data 4: The taxonomy of the representative *nifH* sequences.

Supplementary Data 5: The OTU table of the *nifH* gene sequencing.

Supplementary Data 6: The TPM table of *nifH* gene based on metagenomic analysis.

Supplementary References

81. Wood SN. *Generalized Additive Models: An introduction with R (2nd ed.)*. Boca Raton: Chapman & Hall/CRC press. Taylor & Francis, 2017.

82. Zuur AF, Ieno EN and Walker NJ *et al. Mixed Effects Models and Extensions in Ecology with R*. Dordrecht: Springer, 2009.

83. Carpenter EJ and Capone DG. Nitrogen Fixation in the Marine Environment. In: Capone DG, Bronk DA, Mulholland MR and Carpenter EJ (ed.) *Nitrogen in the Marine Environment* (*Second Edition*). San Diego: Academic Press, 2008, 141–198.

84. Staal M, Meysman FJR and Stal LJ. Temperature excludes N_2-fixing heterocystous cyanobacteria in the tropical oceans. *Nature* 2003; **425**: 504–7.

85. Gallon JR. N_2 fixation in phototrophs: adaptation to a specialized way of life. *Plant Soil* 2001; **230**: 39–48.

86. Berman-Frank I, Cullen J, Shaked Y *et al.* Iron availability, cellular iron quotas, and nitrogen fixation in *Trichodesmium*. *Limnol Oceanogr* 2001; **46**: 1249–60.

87. Danabasoglu G. NCAR CESM2 model output prepared for CMIP6 ScenarioMIP ssp585. Version 20200528. *Earth System Grid Federation* 2019.

88. Kay JE, Deser C and Phillips A *et al.* The community earth system model (CESM) large eensemble project: A community resource for studying climate change in the presence of internal climate variability. *Bull Amer Meteorol Soc* 2015; **96**: 1333–49.

89. Chen Y, Chen Y and Shi C *et al.* SOAPnuke: a MapReduce acceleration-supported software for integrated quality control and preprocessing of high-throughput sequencing data. *GigaScience* 2017; **7**: 1–6.

90. Li D, Liu CM and Luo R *et al.* MEGAHIT: an ultra-fast single-node solution for large and complex metagenomics assembly via succinct *de Bruijn* graph. *Bioinformatics* 2015; **31**: 1674–6.

91. Hyatt D, Chen G-L and LoCascio PF *et al.* Prodigal: prokaryotic gene recognition and translation initiation site identification. *BMC Bioinformatics* 2010; **11**: 119.

92. Huerta-Cepas J, Szklarczyk D and Heller D *et al.* eggNOG 5.0: a hierarchical, functionally and phylogenetically annotated orthology resource based on 5090 organisms and 2502 viruses. *Nucleic Acids Res* 2019; **47**: D309–14.

93. Cantalapiedra CP, Hernández-Plaza A and Letunic I *et al.* eggNOG-mapper v2: Functional annotation, orthology assignments, and domain prediction at the metagenomic scale. *Mol Biol Evol* 2021; **38**: 5825–9.

94. Li W and Godzik A. Cd-hit: a fast program for clustering and comparing large sets of protein or nucleotide sequences. *Bioinformatics* 2006; **22**: 1658–9.

95. Buchfink B, Xie C and Huson DH. Fast and sensitive protein alignment using DIAMOND. *Nat Methods* 2015; **12**: 59–60.

96. Kahlke T and Ralph PJ. BASTA – Taxonomic classification of sequences and sequence bins using last common ancestor estimations. *Methods Ecol Evol* 2019; **10**: 100–3.

97. Langmead B and Salzberg SL. Fast gapped-read alignment with Bowtie 2. *Nat Methods* 2012; **9**: 357–9.

98. Danecek P, Bonfield JK and Liddle J *et al.* Twelve years of SAMtools and BCFtools. *Gigascience* 2021; **10**: giab008.

99. Tarasov A, Vilella AJ and Cuppen E *et al.* Sambamba: fast processing of NGS alignment formats. *Bioinformatics* 2015; **31**: 2032–4.

100. Church MJ, Jenkins BD and Karl DM *et al.* Vertical distributions of nitrogen-fixing phylotypes at Stn ALOHA in the oligotrophic North Pacific Ocean. *Aquat Microbiol Ecol* 2005; **38**: 3–14.

101. Thompson A, Carter BJ and Turk-Kubo K *et al.* Genetic diversity of the unicellular nitrogen-fixing cyanobacteria UCYN-A and its prymnesiophyte host. *Environ Microbiol* 2014; **16**: 3238–49.

102. Church MJ, Short CM and Jenkins BD *et al.* Temporal patterns of nitrogenase gene (*nifH*) expression in the oligotrophic North Pacific Ocean. *Appl Environ Microbiol* 2005; **71**: 5362–70.
